# Correction: An improved GC-MS-SIM analytical method for determination of pendimethalin residue in commercial crops (leaf and soils) and its validation

**DOI:** 10.1371/journal.pone.0340014

**Published:** 2026-01-02

**Authors:** Anindita Paul, Sujan Majumder, L. K. Prasad, M. Sheshu Madhav, Nalli Johnson, K. Padmaja, Satyapriya Singh

There are errors in the Funding statement. The correct Funding statement is as follows: The authors are also thankful to the Council of Scientific & Industrial Research (CSIR) and and ICAR-NIRCA for assistance and for supporting the research work.

The following information is missing from the Acknowledgement statement: The authors are also thankful to the Council of Scientific & Industrial Research (CSIR) for assistance and for supporting the research work.
