# Supplementary material for: An improved GC-MS-SIM analytical method for determination of pendimethalin residue in commercial crops (leaf and soils) and its validation
Source: PLoS One. 2025 Aug 28;20(8):e0328446. doi: 10.1371/journal.pone.0328446 (PMC12393745; doi:10.1371/journal.pone.0328446)
Supplement: S1 Table — (DOCX) [file pone.0328446.s001.docx]

**S1 Table 1. Comparative matrix effect of different combination of clean up dring pendimethalin estimation in tobacco leaf**

| Combination of clean up |  | |
| --- | --- | --- |
|  | **Matrix effect (%)** | **% RSD** |
|  | | |
| 100 mg PSA + 25mg C_18_ + 5mg GCB + 200 mg MgSO_4_ | 46.00 | 3.35 |
| 100 mg PSA + 50 mg C_18_ + 5 mg GCB + 200 mg MgSO_4_ | 34.33 | 6.03 |
| 100 mg PSA + 75 mg C_18_ + 5 mg GCB + 200 mg MgSO_4_ | 14.67 | 2.43 |
| 100 mg PSA + 100 mg C_18_ + 5 mg GCB + 200 mg MgSO_4_ | 12.34 | 8.05 |
| 100 mg PSA + 25 mg C_18_ + 10 mg GCB + 200 mg MgSO_4_ | 40.89 | 3.07 |
| 100 mg PSA + 50 mg C_18_ + 10 mg GCB + 200 mg MgSO_4_ | 44.89 | 2.09 |
| 100 mg PSA + 75 mg C_18_ + 10 mg GCB + 200 mg MgSO_4_ | 32.65 | 3.56 |
| 100 mg PSA + 100 mg C_18_ + 10 mg GCB + 200 mg MgSO_4_ | 10.78 | 2.09 |
| 100 mg PSA + 25 mg C_18_ + 25 mg GCB + 200 mg MgSO_4_ | 36.90 | 1.09 |
| 100 mg PSA + 50 mg C_18_ + 25 mg GCB + 200 mg MgSO_4_ | 26.89 | 0.04 |
| 100 mg PSA + 75 mg C_18_ + 25 mg GCB + 200 mg MgSO_4_ | 17.53 | 3.09 |
| 100 mg PSA + 100 mg C_18_ + 25 mg GCB + 200 mg MgSO_4_ | 9.08 | 4.98 |
| 100 mg PSA + 25 mg C_18_ + 50 mg GCB + 200 mg MgSO_4_ | 25.09 | 2.01 |
| 100 mg PSA + 50 mg C_18_ + 50 mg GCB + 200 mg MgSO_4_ | 20.03 | 0.09 |
| 100 mg PSA + 75 mg C_18_ + 50 mg GCB + 200 mg MgSO_4_ | 10.59 | 6.02 |
| 100 mg PSA + 100 mg C_18_ + 50 mg GCB + 200 mg MgSO_4_ | 12.87 | 3.18 |
| 100 mg PSA + 25 mg C_18_ + 100 mg GCB + 200 mg MgSO_4_ | 10.98 | 3.81 |
| 100 mg PSA + 50 mg C_18_ + 100 mg GCB + 200 mg MgSO_4_ | 4.65 | 2.81 |
| 100 mg PSA + 75 mg C_18_ + 100 mg GCB + 200 mg MgSO_4_ | 4.01 | 1.98 |
| 100 mg PSA + 100 mg C_18_ + 100 mg GCB + 200 mg MgSO_4_ | 2.01 | 2.10 |
