# Supplementary material for: An improved GC-MS-SIM analytical method for determination of pendimethalin residue in commercial crops (leaf and soils) and its validation
Source: PLoS One. 2025 Aug 28;20(8):e0328446. doi: 10.1371/journal.pone.0328446 (PMC12393745; doi:10.1371/journal.pone.0328446)
Supplement: S1 Fig — (a) for solvent standard (b) for tobacco leaf matrix matched standard (c) soil matrix matched standard. (DOCX) [file pone.0328446.s002.docx]

**(a)**

Y = 34331.65X + 13.58649

R^2^ = 0.998

**(b)**

Y = 111111.7X - 426.125

R^2^ = 0.997

**(c)**

Y = 57000.54X - 59.83333

R^2^ = 0.999

**S1. Fig. 1** **Linearity calibration curve**

(a) for solvent standard (b) for tobacco leaf matrix matched standard (c) soil matrix matched standard
