# Supplementary material for: An improved GC-MS-SIM analytical method for determination of pendimethalin residue in commercial crops (leaf and soils) and its validation
Source: PLoS One. 2025 Aug 28;20(8):e0328446. doi: 10.1371/journal.pone.0328446 (PMC12393745; doi:10.1371/journal.pone.0328446)
Supplement: Attachment — Submitted filename: PONE-D-25-08615_reviewer with comments.pdf [file pone.0328446.s003.pdf]

PLOS ONE

An improved GC-MS-SIM analytical method for determination of pendimethalin residue in commercial crops (leaf and soils) and its validation

--Manuscript Draft--

**Manuscript Number:**	PONE-D-25-08615
**Article Type:**	Research Article
**Full Title:**	An improved GC-MS-SIM analytical method for determination of pendimethalin residue in commercial crops (leaf and soils) and its validation
**Short Title:**	A rugged novel analytical tool for exporting commercial crops with less residual load
**Corresponding Author:**	Anindita Paul, PhD<br>Central Tobacco Research Institute<br>Rajahmundry, INDIA
**Keywords:**	Pendimethalin residue; Tobacco; GC-MS; SIM; Ethyl acetate extraction; method development
**Abstract:**	Pendimethalin as pre-emergence herbicide has been extensively used globally. Pendimethalin is generally used as pre-emergence herbicide cum suckericide in different growing region of Flue-Cured Verginia (FCV) tobacco by applying indiscriminately in irrigation channel. In quest of safety regulations related to pendimethalin residues in cured leaf tobacco and tobacco growing soils, a rapid and robust method is developed by GC-MS single quadruple system. The GC-MS single ion monitoring (SIM) analytical method developed to monitor the pendimethalin residues in tobacco below the guidance residue levels (GRL) or not. The GC-MS single ion monitoring (SIM) analytical method, achieved good linearity (R2>0.99) with LOD and LOQ values of 0.001 mg/kg and 0.005 mg/kg, respectively. The method gave more than 80% recovery (5% RSD) showed compliance with international specification of DG-SANTE guidelines. In the majority of samples, pesticide residue levels were below the guidance residue levels (GRL) value set by Center for Scientific Research Relative to Tobacco (CORESTA). The analytical method developed for true detection of pendimethalin residue at very trace levels with acceptable recovery level and matrix effect. Looking at the consumer safety the method can be used in monitoring the pendimethalin residues in FCV tobacco and FCV tobacco grown soils at regular intervals.
**Order of Authors:**	Anindita Paul, PhD
	Sujan Majumder
	L. K. Prasad
	M. Sheshu Madhav
	Nalli Johnson
	K. Padmaja
	Satyapriya Singh
**Additional Information:**	
**Question**	**Response**
**Financial Disclosure**<br><br>Enter a financial disclosure statement that describes the sources of funding for the work included in this submission. Review the submission guidelines for detailed requirements. View published research articles from *PLOS ONE* for specific	The author(s) received no specific funding for this work.

Powered by Editorial Manager® and ProduXion Manager® from Aries Systems Corporation

examples.<br><br>This statement is required for submission and **will appear in the published article** if the submission is accepted. Please make sure it is accurate.<br><br>**Funded studies**<br>Enter a statement with the following details:<br>• Initials of the authors who received each award<br>• Grant numbers awarded to each author<br>• The full name of each funder<br>• URL of each funder website<br>• Did the sponsors or funders play any role in the study design, data collection and analysis, decision to publish, or preparation of the manuscript?<br><br>Did you receive funding for this work?	
**Competing Interests**<br><br>Use the instructions below to enter a competing interest statement for this submission. On behalf of all authors, disclose any competing interests that could be perceived to bias this work—acknowledging all financial support and any other relevant financial or non-financial competing interests.<br><br>This statement is required for submission and **will appear in the published article** if the submission is accepted. Please make sure it is accurate and that any funding sources listed in your Funding Information later in the submission form are also declared in your Financial Disclosure statement.<br><br>View published research articles from *PLOS ONE* for specific examples.	NO authors have competing interests

**NO authors have competing interests**<br><br>Enter: *The authors have declared that no competing interests exist.*<br><br>**Authors with competing interests**<br><br>Enter competing interest details beginning with this statement:<br><br>*I have read the journal's policy and the authors of this manuscript have the following competing interests: [insert competing interests here]*<br><br>* typeset	
**Ethics Statement**<br><br>Enter an ethics statement for this submission. This statement is required if the study involved:<br><br>• Human participants<br>• Human specimens or tissue<br>• Vertebrate animals or cephalopods<br>• Vertebrate embryos or tissues<br>• Field research<br><br>Write "N/A" if the submission does not require an ethics statement.<br><br>General guidance is provided below. Consult the submission guidelines for detailed instructions. **Make sure that all information entered here is included in the Methods section of the manuscript.**	N/A

**Format for specific study types**<br><br>**Human Subject Research (involving human participants and/or tissue)**<br>• Give the name of the institutional review board or ethics committee that approved the study<br>• Include the approval number and/or a statement indicating approval of this research<br>• Indicate the form of consent obtained (written/oral) or the reason that consent was not obtained (e.g. the data were analyzed anonymously)<br><br>**Animal Research (involving vertebrate animals, embryos or tissues)**<br>• Provide the name of the Institutional Animal Care and Use Committee (IACUC) or other relevant ethics board that reviewed the study protocol, and indicate whether they approved this research or granted a formal waiver of ethical approval<br>• Include an approval number if one was obtained<br>• If the study involved *non-human primates*, add *additional details* about animal welfare and steps taken to ameliorate suffering<br>• If anesthesia, euthanasia, or any kind of animal sacrifice is part of the study, include briefly which substances and/or methods were applied<br><br>**Field Research**<br><br>Include the following details if this study involves the collection of plant, animal, or other materials from a natural setting:<br>• Field permit number<br>• Name of the institution or relevant body that granted permission	
**Data Availability**<br><br>Authors are required to make all data underlying the findings described fully available, without restriction, and from the time of publication. PLOS allows rare exceptions to address legal and ethical concerns. See the PLOS Data Policy and FAQ for detailed information.	Yes - all data are fully available without restriction

A Data Availability Statement describing where the data can be found is required at submission. Your answers to this question constitute the Data Availability Statement and **will be published in the article**, if accepted.<br><br>**Important:** Stating 'data available on request from the author' is not sufficient. If your data are only available upon request, select 'No' for the first question and explain your exceptional situation in the text box.<br><br>Do the authors confirm that all data underlying the findings described in their manuscript are fully available without restriction?	
**Describe where the data may be found in full sentences. If you are copying our sample text, replace any instances of XXX with the appropriate details.**<br><br>• If the data are **held or will be held in a public repository**, include URLs, accession numbers or DOIs. If this information will only be available after acceptance, indicate this by ticking the box below. For example: *All XXX files are available from the XXX database (accession number(s) XXX, XXX.*).<br>• If the data are all contained **within the manuscript and/or Supporting Information files**, enter the following: *All relevant data are within the manuscript and its Supporting Information files.*<br>• If neither of these applies but you are able to provide **details of access elsewhere**, with or without limitations, please do so. For example:<br><br>*Data cannot be shared publicly because of [XXX]. Data are available from the XXX Institutional Data Access / Ethics Committee (contact via XXX) for researchers who meet the criteria for access to confidential data.*<br><br>*The data underlying the results presented in the study are available from (include the name of the third party*	NA

and contact information or URL).<br>• This text is appropriate if the data are owned by a third party and authors do not have permission to share the data.<br><br>* typeset	
Additional data availability information:	

Manuscript
Click here to access/download;Manuscript;Paul A_corrected.docx

An improved GC-MS-SIM analytical method for determination of pendimethalin residue in commercial crops (leaf and soils) and its validation

Anindita Paul[1*†], Sujan Majumder[2†], L. K. Prasad[1], M. Sheshu Madhav[1], Nalli Johnson[1†], K. Padmaja[1], and Satyapriya Singh[3]

[1] *ICAR-Central Tobacco Research Institute, Rajahmundry 533105, Andhra Pradesh, India*

[2]*ICAR-Indian Institute of Vegetable Research, Varanasi- 221305, India*

[3]*Central Horticultural Experiment Station (ICAR-IIHR), Bhubaneswar, Odisha-751019, India*

[†]The authors contributed equally

*Corresponding author: aninditapaul20@gmail.com.

Abstract

Pendimethalin as pre-emergence herbicide has been extensively used globally. Pendimethalin is generally used as pre-emergence herbicide cum suckericide in different growing region of Flue-Cured Verginia (FCV) tobacco by applying indiscriminately in irrigation channel. In quest of safety regulations related to pendimethalin residues in cured leaf tobacco and tobacco growing soils, a rapid and robust method is developed by GC-MS single quadruple system. The GC-MS single ion monitoring (SIM) analytical method developed to monitor the pendimethalin residues in tobacco below the guidance residue levels (GRL) or not. The GC-MS single ion monitoring (SIM) analytical method, achieved good linearity (R^2>0.99) with LOD and LOQ values of 0.001 mg/kg and 0.005 mg/kg, respectively. The method gave more than 80 % recovery (5 % RSD) showed compliance with international specification of DG-SANTE guidelines. In the majority of samples, pesticide residue levels were below the guidance residue levels (GRL) value set by Center for Scientific Research Relative to Tobacco (CORESTA). The analytical method developed for true detection of pendimethalin residue at very trace levels with acceptable recovery level and matrix effect. Looking at the consumer safety the method can be used in monitoring the pendimethalin residues in FCV tobacco and FCV tobacco grown soils at regular intervals.

Keywords: Pendimethalin residue; Tobacco; GC-MS; SIM; Ethyl acetate extraction; method development

Introduction

Tobacco (*Nicotiana tabacum* L.), one of the world's leading commercial crops, is grown extensively in countries such as India, China, Brazil, USA, etc. Tobacco is being cultivated in an area of about 0.47 million hectares, accounting for 0.33 per cent of the total arable land in the country (http://www. fao.org/faostat/en/#compare). The increasing demand for the Indian tobacco made the crop to be the major revenue source to India with an export value of US$ 859.45 million. India is maintaining its dominant position in the world as third largest producer of tobacco (~800 million kg) after China and Brazil . In countries like India, with tropical-humid climate, the incidences of pest and disease infestations are frequent and application of pesticides for their management is almost obligatory. The pesticides applied to tobacco during its cultivation may remain in the leaf till harvesting and even after curing/ post-harvest processing in the final manufactured product.[1-3] While smoking, the pyrolytic products from tobacco plant matrix may interact with the pesticides or with the pyrolytic products of pesticides and results in the formation of more toxic smoke than that from the sole pesticide residues which is being inhaled by mainstream smoker and several other passive smokers. [4] Clapp and Shelar (1972) reported that rate of transfer of pesticides from tobacco into smoke averaged about 12% of that in the tobacco before combustion.[5] Transfer of suckericide like pendimethalin residue to plant leaf system may reach up to 20 % of the residues in the unsmoked tobacco as this pesticide is mainly applying indiscriminately in irrigation channel at 90 days after planting which get translocated to soil and plant system very rapidly.[6-8] Looking into the both the apprehensions related to accumulation of pesticide residues at toxic levels in the final produce drives the safety regulations to become more and more stringent in most countries. Despite several awareness campaigns about the imminent potential health problems associated with tobacco, millions of people, particularly in lower and middle-income societies still indulge in cigarette smoking in this global COVID 19

pandemic. [9] Presence of pesticide residues in tobacco further aggravates the health risk not only to the smoker, but also to those subject to passive inhalations. Thus, monitoring of pesticide residues in tobacco is an important issue of critical concern from public health and safety point of view demanding implementation of stringent regulatory policies. [10]

The Guidance Residue Levels (GRL) set by Cooperation Center for Scientific Research Relative to Tobacco[10], list for tobacco contains different classes of pesticides, such as organochlorine, organophosphorus, pyrethroids etc. Pendimethalin, a pesticide of the dinitroaniline class, is widely used as an herbicide in tobacco cultivation across the world, including India. However, its use as a suckericide for sucker control in tobacco has drawn attention of the scientific community as well as of the exporting agencies. Hence, determination of pendimethalin residue is an urgent need as there is no report on Indian tobacco.

The complex nature of tobacco matrix, led many obstacles during sample preparations and method accuracy very less while doing analysis by GC.[11-13] Very few literature is available in pertaining pendimethalin residue analysis in tobacco matrix with selective determination by GC MS.[14] The previously developed method owned time consuming acquisition and unnecessarily incurred the costing of method analysis and more over the quantification limit is higher than 0.01 mg/kg which demands the improvisation of the existing method in compliances with GRL (Guidance Residue level) to target the trade barrier associated with tobacco.

The objective of the present study was to develop a sensitive, effective, and economic analytical method for pendimethalin pesticide in tobacco using a GC-MS single quadrupole instrument. In addition to the optimization of the selected ion monitoring (SIM) parameters, the technique of high-resolution GC-MS is demonstrated in resolving complex matrix effect problems.

Experimental

Collection of tobacco samples

The cured leaf samples of Flue Cured Virginia (FCV) tobacco were collected from Andhra Pradesh and Karnataka, the two major tobacco producing states of India. In all 60 representative leaf and soil samples were drawn from major FCV tobacco growing districts i.e *Nellore* (14.44° N 79.99° E), *Prakasam* (15.50° N 80.05° E), *West Godavari* (16.57° N 82.15° E) of Andhra Pradesh and Mysore (12.08° N 76.32° E) district of Karnataka. After removing the midribs of the representative leaf samples, around 50 g of leaf lamina were oven dried at 60° C for 2 h. The dried leaves were powdered, homogenized, passed through 1-mm sieve, and utilized for further analyses. The soil samples were air-dried, homogenised and passed through 2 mm sieve for further analyses.

Chemicals and apparatus

The certified reference standard of pendimethalin (98.9 % purity) was procured from M/S Sigma-Aldrich Pvt. Ltd., India. Ethyl acetate of analytical grade - the extraction solvent, anhydrous sodium sulphate (Na_2SO_4), magnesium sulphate ($MgSO_4$) and glacial acetic acid (Analytical reagent grade) were procured from Thomas Baker (Mumbai, India). GC-MS grade ethyl acetate was procured from Merck (Mumbai, India). The adsorbents, Primary secondary amine (PSA), graphitized carbon black (GCB) and C_{18} were procured from Agilent Technologies, Bangalore, India. Precision balance (Vibra, Adair Dutt, Mumbai, India), vortex mixer (Spinix, Borosil, Mumbai, India), micro centrifuge (Borosil R Mumbai, India), centrifuge (Kubota, Germany) and ultra sonicator bath (Oscar electronics, Mumbai, India) were used while preparation of samples and reagents.

Preparation of standard solutions and matrix matched standards

The standard stock solutions of pendimethalin were prepared by weighing 10 (± 0.01) mg of the reference standards dissolving in 10 mL ethyl acetate in a certified 'A' class volumetric

flask. The resulted final concentration of ~ 1000 µg/mL stored in dark in a refrigerator at -20 (±2) ^{o}C. An intermediate working standard mixture of 10 µg/mL was prepared by mixing appropriate quantities of the standard stock in ethyl acetate constancy of the working standard solution was checked against freshly prepared working standards (1 µg/mL) from the intermediate standards as per SANTE /11312/2021 guidelines.[15] From the 1 µg/mL working standard solution different calibration standards (0.001-0.08 µg/mL) were prepared by serial dilution with ethyl acetate. The matrix matched standards at the same concentration were prepared by extracting control tobacco matrix and soils and spiking the extract with appropriate volumes of the working standard solutions.

Procedure of Extraction and clean-up

The entire sample were mixed thoroughly and selected randomly for further analysis for both tobacco leaf powder and soil. The samples were extracted by following earlier reported method with minor modifications.[16] Samples of 20 g homogenate (2 g tobacco + 18 mL water containing 0.5 % acetic acid) were extracted by 10 mL ethyl acetate (vortex for 1 min) followed by the addition of 10 g Na_2SO_4 (vortex for 1 min). This was subjected to phase separation by centrifugation at 5000 rpm for 5 min. A supernatant of 2 mL ethyl acetate was cleaned up using two different combinations of dispersive solid-phase extraction (*d*SPE) sorbents as follows: 25, 50, 75 and 100 mg of PSA per mL of supernatant. Thereafter the combinations of C_{18} and GCB with $MgSO_4$,were evaluated to get the good and acceptable results. The supernatant of the above extract (1 mL) was centrifuged at 10000 rpm for 5 min. It was further filtered through a 0.2 µm polytetrafluoroethylene (PTFE) membrane filter and finally injected (1 µL) into the GC-MS system and analysed in SCAN mode with reference standards (based on retention time) and in SIM mode with quantifier and qualifier ions (*m/z*). Soil samples were also extracted using the same aforementioned sample preparation workflow.

Instrumentation

The GC-MS QP-2010 Plus (single quadrupole, Shimadzu Corporation, Kyoto, Japan) system was equipped with ZB-5 (5% diphenyl, 95% dimethylpolysiloxane, 30 m (l) x 0.25 mm (id), 0.1 µm film thickness) capillary column and autosampler. The GC-MS separation of Pendimethalin was achieved by an optimized oven temperature program that started from an initial temperature of 90 °C (hold for 0.5 min), ramped at the rate of (@) 20 °C min^{-1} up to 180 °C (hold 1 min), increased to 240 °C @ 12 °C min^{-1} (hold for 1 min), again increased to 260°C @ 15 °C min^{-1} (hold for 1 min) and finally increased up to 280°C @ 12 °C min^{-1} (hold 0.5 min). This program resulted in total run-time of 16.5 min. The sample solutions were injected in split injection mode (split ratio 10 and pressure 29.1 psi for 1 min) with the injection volume of 1 µL. The injector temperature was set at 250°C. The ion source temperature was 200°C and the interface was at 280°C. The detector voltage was set at 0.87 kV and the data acquisition was carried out in the selected ion monitoring (SIM) mode with specific *m/z* ions for selective identification of pendimethalin. Ultra-pure (99.999 %) grade helium (INOX Limited, Hyderabad) was used as the carrier gas. The flow rate of Helium was maintained as 3.14 mL/min with a linear velocity of 65.6 cm/sec. The mass spectrometer was operated using electron impact ionization (EI, 70 eV).

Method performance

A single laboratory based method validation was performed as per SANTE/11312/2021 guideline which includes linearity, limit of detection (LOD), limit of quantification (LOQ), matrix effect, accuracy and repeatability and recovery. The calibration curves for linearity establishment for pendimethalin in pure solvent and in matrix (tobacco leaf and soil matrix) by establishing seven levels ranging between 0.001-0.08 mg/L (Supplementary Figure 1). The sensitivity of the method was determined in terms of limit of detection (LOD) and limit of quantification (LOQ) which decides as the smallest measured quantity in tobacco leaf and

soil matrix at which the signal to noise ratio (S/N) were 3:1 and 10:1, respectively. The method LOQ was the lowest concentration at which method was validate with acceptable recoveries. Recovery of the pendimethalin from tobacco leaf and soil matrix was studied at 0.01, 0.02, 0.05, 0.10 and 0.50 mg/kg with six replicates each (Table 3). The average recoveries within the range of 80-120 % are accepted for both the matrices (tobacco leaf and soil) as per SANTE/11312/2021 guideline.[15]. The matrix effect was determined by spiking untreated extract with the targeted pesticide at 0.05 mg/kg level. The peak area response of pendimethalin in ethyl acetate solvent was compared with that of the corresponding peak area in the matrix matched standard at the same concentration.

Matrix effect (ME%) = [(peak area of the matrix standard - peak area of the solvent standard)/peak area of the solvent standard] × 100.

A positive or negative value of ME (%) indicates that matrix induced signal enhancement and suppressions respectively. [17-18]

Analysis of incurred samples:

We have collected FCV tobacco samples from farmers' field of Nellore, Prakasam and West Godavari district of Andhra Pradesh and estimated pendemethalin residues using above mentioned standardized extraction and analytical method.

Results and discussion

Optimization of extraction and clean up method

Extraction of targeted compounds from the tobacco matrix is very challenging task. [16] The ethyle ecetate were selected for the extraction of the targeted compounds, which provided accepted results in the previously reported studies (19,20). However, acetic acid is generally used to stabilize several natural compounds present in tobacco matrix during sample preparation which interferes in the response of pendimethalin as a false positive. [16,19] I, the extract was injected without cleanup and as a results the yellow color pigments of tobacco

leaf extract was highly reducing the recovery percentage of pendimethalin of about 2.5-3%. On the other hand it was causing interference in GC-liner which forced to change the liner frequently. Therefore, cleanup plays a key role in sample preparation in case of complex tobacco matrix. To reduce the matrix induced signal enhancement, the ethyl acetate extract of tobacco leaf and soil matrix was undergone to clean up process with PSA, which provided lower matrix effect up to some extent (12.34%) with 100 mg of PSA + 200 mg $MgSO_4$. However to further reduce the matrix effect and matrix interference some combination were tried with 100 mg PSA + (25, 50, 75 and 100 mg) C_{18} + (5, 10, 25, 50 and 100 mg) GCB alone with 200 mg of $MgSO_4$ (Supplementary table). which can reduce the matrix effect to less than17%. Though initially, with 100 mg GCB gave charcoal interference during injection whereas lesser than 50 mg GCB gave more colored solution resulting ghost peaks along with target compounds with matrix effect upto 46%. Finally, the combination of 100 mg PSA, 100 mg C_{18}, 50 mg GCB and 200 mg $MgSO_4$ provided the lowest matrix effect for the targeted compound and also which helped to reduce frequent cleaning as well as change of GC-Liner.

Hence cleanup of 2 mL ethyl acetate extract of tobacco leaf and soil matrix were optimized with 100 mg PSA, 100 mg C_{18}, 50 mg GCB and 200 mg $MgSO_{4.}$ The one of the novelty of this study is as tobacco is a complecated matrix and it is very difficult to have lesser matrix effect of targeted compound, since the extraction method is need to be optimized along with analytical parameters which is discussed below.

This method provided accepted recovery for tobacco leaf ranging from 87-95.34% with 2.73-8.15% RSD and in case of soil 80.01-84.99% with 1.42-6.25% RSD which complies to the analytical quality control guideline. [10,15]

Confirmative analysis by GC-MS:

The probabilities of acquiring false detection in plant or soil matrices become very crucial as they may act as interfering components in deferring the response of target pesticide at the retention time while using GC-MS instrument. Hence to deescalate the false detection, a novel GC-MS based selective ion monitoring (SIM) method has developed employing with different acquisition parameters such as confirmative identification based on the quantifier-qualifier ions (m/z) ratio. Depending on the molecular fragmentation recorded in the mass detection system, four ions (*m/z*) namely, 162, 191, 252 and 281 were selected. However, the reference and target ion combination resulted a complex chromatogram where, the base ion, 162, can exist in several other molecules. Hence, the ion 162 was ejected as base ions and m/z 252 was selected as quantifier ion and rest four ions (162, 191, 208, 252, and 281) were selected as qualifier ions (Fig. 3a, 3b and 4) for selective identification of pendimethalin. It was observed that selection of 252 ion as a base peak improved the identification and quantification of pendimethalin as compared to 162 ion. The developed method was employed for analysis of some field samples (Fig. 5). The results of Table 1 and 2 indicated that the pendimethalin residues detected in both tobacco leaf and soil are in the range of 0.0052-0.0169 mg kg^{-1} which are far below than GRL level (5 mg kg^{-1}) of pendimethalin in tobacco (8). All the samples achieved precision >91% with good repeatability.

Comparative performance with previously developed method:

Earlier approaches of pendimethalin estimation in complecated matrix like peanut, tobacco involves greater amount of clean up reagents which not only incurred more cost but also induces indirect matrix intereferences (10-34%). [19,22-23] More over the GC-MS based methods also not so robust such as the acqusition parameters are time consuming, the transitions were not well defined. More over, the LOQ levels are higher upto 0.01 mg/kg which in turns suggest the redevelopment of pendimethalin estimation in tobacco like complicated matrix. On the other hand, pendimethalin residues in the incurred samples were

not estimated earlier. [24] The present study shaded light into these drawbacks and re developed a new method for estimating pendimethalin residues in both leaf and soil to interrogate the residues present in incurred samples and as well as the acquisition method is faster and robust having LOQ level of 0.005 mg/kg for both leaf and soil matrices. The developed method itself given an insight of fate of utilization of higher GCB and C18 and it was further optimized to obtain better recovery% and lower matrix effect. In this way, the developed method stands out than the existing method to estimate pendimethalin in complex matrices.

Method validation

LOD, LOQ and recovery:

The analytical method for estimation of pendimethalin residues in tobacco leaf and soil was validated according to SANTE guideline. Pendimethalin had a retention time (Rt) of 10.37 min in newly developed analytical method. The good linearity achieved with coefficient of determination (R^2) value of 0.998 for solvent standard, 0.997 for tobacco leaf matrix matched standard and 0.999 for soil matrix matched standard within the calibration range of 0.001- 0.08 mg/kg. The LOQ for pendimethalin were established as and 0.005 mg/kg for both the matrices, which were far below the GRL value (5 mg/kg). The percentage recovery was estimated at five levels for both tobacco leaf as well as soil matrices separately. The percentage recoveries at 0.01, 0.02, 0.05, 0.10 and 0.50 mg/kg were found in the range between 80 -95 % for both the matrices (Table 3) with relative standard deviation (% RSD) less than 20 % which met internationally accepted SANTE guidelines. The average matrix effect (ME) percentage were less than ~10 % for leaf and ~11 % for soil matrix. Off late, Paul et al, 2021 had established a precise liquid chromatographic with high resolution mass spectrometry (Orbitrap) method for detection of multiple pesticide residues in tobacco matrix and achieved good screening detection limit at 5 ng/g level which fulfilled the requirements

of analytical quality control guideline of SANTE/11312./2021.[15] Off late, Rahman et al. (2012) and Chen et al., 2013 developed a high performance liquid chromatography (HPLC) method in detecting some pesticides with only single acquisition parameter. Particularly, many plant matrix compound can elute at same retention time with that of same target pesticide called false detection and the concentration of plant matrix interfering compound is more prevalent than the target pesticide. To resolve the problem mass fragmentation can give the confirmative analysis to eject the falsified detection induced by matrices. [20-21].

Analysis of incurred samples:

The analysed samples found to have no residues of pendimethalin as residues estimated ~~below detection limit and~~ well below the GRL (5 mg/kg) which clearly indicated that the farmers of those representative districts of Andhra Pradesh are following good agricultural practices.

Conclusions

The novel analytical method developed for true detection of pendimethalin residue at very trace levels which satisfy the internationally accepted guidelines with acceptable recovery level and matrix effect. The new method achieved good linearity (R^2>0.99) over the concentration range of 0.001-0.08 mg kg^{-1} had limit of determination (LOD) and limit of quantification (LOQ) values of 0.001 mg kg^{-1} and 0.005 mg kg^{-1}, respectively. The method with more than 80% recovery (with 5% RSD). The method ensures sensitive and accuriate detection of pendimehalin residues in tobacco leaf and soil samples. The studies showed that the majority of samples, contain pendimethalin pesticide residue levels far below the GRL value set by CORESTA. The ethyl acetated based extraction method resulted good recovery with good repeatability. It also shows that the levels of pendimethalin residues in FCV tobacco are very low to negligible level and pendimethalin used across FCV tobacco as suckericide does not result residue above critical level. It also holds promise in facilitating

selective and sensitive residue analyses of pesticides in such a complicated matrix like tobacco and resolving false detection. This method is suitable and will be applicable for routine analysis of pesticide residues in tobacco samples as well as soil samples. Looking at the consumer safety the method can be used in monitoring the pendimethalin residues in FCV tobacco and FCV tobacco grown soils at regular intervals.

Acknowledgements

The authors are thankful to Director, ICAR-CTRI, Rajahmundry for support to carry out this research.

Conflicts of Interest

All authors declare no conflict of interest

Availability of data

The data that support the findings of this study are available from the corresponding author at reasonable request.

References

[1] Morris, P. Chlorinated pesticide residues in Australian cigarettes. **1972,** http://legacy.library.ucsf.edu/tid/rvj35e00. Accessed 20 October 2022.

[2] https://tobaccoboard. com/tbdata/publicationsfiles/AR _ 2020 21 _ Eng.pdf accessed on 21July, 2022

[3] Clark, T.; Kaussmann, E.; Römer, E.; Schepers, G.; The fate of imidacloprid in tobacco smoke of cigarettes made from imidacloprid-treated tobacco. *Pestic. Sci.* **1998.** *52*(2), 119-125.

[4] Lorenz, W.; Bahadir, M.; Korte, F. Thermolysis of pesticide residues during tobacco smoking. *Chemosphere*. **1987.** *16*(2), 521–522.

[5] Clapp, W. L.; Shelar, G. R. The determination of chlorinated pesticides in mainstream smoke. (**1972**). http://legacy.library.ucsf.edu/tid/cdv69d00. Accessed on 25 October 2022.

[6] Bowery, T. G.; Gatterdam, P. E.; Guthrie, F. E.; Rabb, R. L. Metabolism of insecticide residues, fate of inhaled C^{14}TDE in rabbits. *J. Agric. Food Chem.* **1965.** (4), 356–359.

[7] Atallah, Y. H.; Dorough, H. W. Insecticide residues in cigarette smoke: transfer and fate in rats. *J. Agric. Food Chem.* **1975**. 23(1), 64–71.

[8] Rodgman, A.; Perfetti, T. A. The Chemical Components of Tobacco and Tobacco Smoke **2013.** Florida: CRC Press, Taylor & Francis Group.

[9] WHO report on the global tobacco epidemic, **2021**. Warning about the dangers of tobacco. https://www.who.int/news-room/fact-sheets/detail/tobacco (accessed August 08, 2021).

[10] CORESTA GUIDE N° 1, Agrochemical guidance residue levels. 2019. https://www.coresta.org/sites/default/files/technical_documents/main/Guide-No01-GRLs5th-Issue-Oct20.pdf(accessed on 24.12.2021).

[11] Haib, J.; Hofer, I.; Renaud, J. M. Analysis of multiple pesticide residues in tobacco using pressurized liquid extraction, automated solid-phase extraction clean-up and gas chromatography–tandem mass spectrometry. *J. Chromatogr. A.* **2003.** *1020*(2), 173-187.

[12] Cai, J.; Gao, Y.; Zhu, X.; Su, Q. Matrix solid phase dispersion–Soxhlet simultaneous extraction clean-up for determination of organochlorine pesticide residues in tobacco. *Anal. Bioanal. Chem.* **2005.** *383*(5), 869-874.

[13] Chen, X.; Bian, Z.; Hou, H.; Yang, F.; Liu, S.; Tang, G.; Hu, Q. Development and validation of a method for the determination of 159 pesticide residues in tobacco by gas chromatography–tandem mass spectrometry. *J. Agric. Food Chem.* **2013.** *61*(24), 5746-5757.

[14] Cai, J.; Liu, B.; Zhu, X.; Su, Q. Determination of pyrethroid residues in tobacco and cigarette smoke by capillary gas chromatography. *J. Chromatogr. A.* **2002.** *964*(1-2), 205-211.

[15] SANTE/11312/2021. Analytical quality control and method validation procedures for pesticides residues analysis in food and feed, (2021) https://ec.europa.eu/food/sites/food/files/plant/docs/pesticides_mrl_guidelineswrkdoc_11945.pdf (accessed on 02.02.2022).

[16] Paul A.; Khan Z.; Bhattacharyya A.; Majumder S.; Banerjee K. Multiclass pesticide residue analysis in tobacco (*Nicotiana tabacum*) using high performance liquid chromatography-high resolution (Orbitrap) mass spectrometry: A simultaneous

screening and quantitative method. *J. Chromatogr. A.* **2021.** *1648*:462208, doi.: https://doi.org/10.1016/j.chroma.2021.462208

[17] Hingmire S.; Oulkar D. P.; Utture S. C.; Shabeer A. T. P.; Banerjee K. Residue analysis of fipronil and difenoconazole in okra by liquid chromatography tandem mass spectrometry and their food safety evaluation. *Food Chem.* **2015.** *17*: 145–151.

[18] Shinde, R.; Pardeshi, A.; Dhanshetty, M.; Anastassiades, M.; Banerjee, K. Development and validation of an analytical method for the multiresidue analysis of pesticides in sesame seeds using liquid-and gas chromatography with tandem mass spectrometry. *J. Chromatogr. A.* **2021.** *1652,* 462346. https://doi.org/10.1016/j.chroma.2021.462346

[19] Ghosh, R. K.; Khan, Z. S.; Banerjee, K.; Reddy, D. D.; Johnson, N.; Ray, D. P. Elucidation of False Detection of Pesticides During Residue Analysis in Indian Tobacco by Multidimensional GC-MS. *J. AOAC Int.* **2020.** *103*(1), 55-61.

[20] Chen, X.; Bian, Z.; Hou, H.; Yang, F.; Liu, S.; Tang, G.; Hu, Q. Development and validation of a method for the determination of 159 pesticide residues in tobacco by gas chromatography–tandem mass spectrometry. *J. Agric. Food Chem.* **2013.** *61*(24), 5746-5757.

[21] Rahman, M. A.; Chowdhury, A. Z.; Moniruzzaman, M.; Gan, S. H.; Islam, M. N.; Fardous, Z.; Alam, M. K. Pesticide residues in tobacco leaves from the Kushtia district in Bangladesh. *Bull. Environ. Contam. Toxicol.* **2012.** *89*(3), 658-663.

[22] Saha, A.; Shabeer T.P.A.; Banerjee, K., Hingmire, S.; Bhaduri, D.; Jain, N. K.; Utture, S. Simultaneous analysis of herbicides pendimethalin, oxyfluorfen, imazethapyr and quizalofop-p-ethyl by LC–MS/MS and safety evaluation of their harvest time residues in peanut (Arachis hypogea L.). *J. Food Sci. Technol.* **2015.** *52*(7), 4001-4014.

[23] Majumder, S.; Mandal, S.; Majumder, B.; Paul, A.; Paul, T.; Sahana, N.; Mondal, P. A liquid chromatographic method for determination of acetamiprid and buprofezin residues and their dissipation kinetics in paddy matrices and soil. *Environ. Sci. Pollut. Res.* **2022.** *29*(1), pp.1401-1412. https://doi.org/10.1007/s11356-021-15784-4

[24] Ghosh, R. K.; Reddy, D. D.; Johnson, N.; Mahadevaswamy, M.; Bindu, J. P. GC-MS/SIM analytical method for determination of pendimethalin residue in tobacco matrix. *Tobacco Research.* **2016.** Indian Society of Tobacco Science

Table 1. Pendimethalin residues present in FCV tobacco leaf samples analyzed by GC-MS/SIM method.

Sample Details	Location	Pendimethalin concentration (mg kg^{-1})
**SLS Tobacco 22**	Podili, (15.6070° N, 79.6146° E) Prakasam district of Andhra Pradesh	0.00817
**SLS Tobacco 23**	Podili, (15.6070° N, 79.6146° E) Prakasam district of Andhra Pradesh	0.00912
**SLS Tobacco 25**	Kandukur, (15.2197° N, 79.9025° E) Prakasam district of Andhra Pradesh	0.00324
**SLS Tobacco 26**	Kandukur, (15.2197° N, 79.9025° E) Prakasam district of Andhra Pradesh	0.00771
**SLS Tobacco 27**	Kandukur, (15.2199° N, 79.9027° E) Prakasam district of Andhra Pradesh	0.00524
**SLS Tobacco 28**	Kaligiri, (14.8279° N, 79.6937° E) Prakasam district of Andhra Pradesh	0.00976
**SLS Tobacco 29**	D C Palli, (14.6876° N, 79.5034° E) Prakasam district of Andhra Pradesh	BDL
**SLS Tobacco 30**	D C Palli, (14.6876° N, 79.5034° E) Prakasam district of Andhra Pradesh	BDL
**SLS Tobacco 32**	Kanigiri, (15.4048° N, 79.5067° E) Prakasam district of Andhra Pradesh	BDL
**SLS Tobacco 33**	Kanigiri, (15.4048° N, 79.5067° E) Prakasam district of Andhra Pradesh	BDL
**SLS Tobacco 35**	Kanigiri, (15.4048° N, 79.5067° E) Prakasam district of Andhra Pradesh	0.00987
**NLS Tobacco 32**	Jangareddygudem, (17.1223° N, 81.2923° E) West Godavari district of Andhra Pradesh	0.00859
**NLS Tobacco 17**	Devarapalli, (17.0350° N, 81.5624° E) West Godavari district of Andhra Pradesh.	0.01691
**NLS Tobacco 30**	Koyyalagudem, (17.4521° N, 81.6528° E) West Godavari district of Andhra Pradesh	BDL
**NLS Tobacco 32**	Jangareddygudem, (17.1223° N, 81.2923° E) West Godavari district of Andhra Pradesh	BDL

**NLS Tobacco 33**	Jangareddygudem, (17.1223° N, 81.2923° E) West Godavari district of Andhra Pradesh	0.00505
**NLS Tobacco 34**	Jangareddygudem, (17.1223° N, 81.2923° E) West Godavari district of Andhra Pradesh	0.00505
**NLS Tobacco 36**	Koyyalagudem, (17.4521° N, 81.6528° E) West Godavari district of Andhra Pradesh	0.00305
**NLS Tobacco 37**	Jangareddygudem, (17.1223° N, 81.2923° E) West Godavari district of Andhra Pradesh	BDL
**NLS Tobacco 38**	Jangareddygudem, (17.1223° N, 81.2923° E) West Godavari district of Andhra Pradesh	BDL
**NLS Tobacco 40**	Koyyalagudem, (17.4521° N, 81.6528° E) West Godavari district of Andhra Pradesh	BDL
**NLS Tobacco 42**	Koyyalagudem, (17.4521° N, 81.6528° E) West Godavari district of Andhra Pradesh	BDL
**KLS Tobacco 5**	Periyapatna, (12.3384° N, 76.0965° E) Mysore district of Karnataka district of Karnataka.	0.00501
**KLS Tobacco 4**	Periyapatna, (12.3384° N, 76.0965° E) Mysore district of Karnataka district of Karnataka.	0.00842
**KLS Tobacco 7**	Ramnathapura, (12.6166° N, 76.0842° E) Mysore district of Karnataka.	BDL
**KLS Tobacco 3**	Hunsur, (12.3091° N, 76.2833° E) district Mysore of Karnataka	BDL
**KLS Tobacco 63**	Ramnathapura, (12.6166° N, 76.0842° E) Mysore district of Karnataka.	0.00406
**KLS Tobacco 64**	Ramnathapura, (12.6166° N, 76.0842° E) Mysore district of Karnataka.	0.00506
**KLS Tobacco 65**	Ramnathapura, (12.6166° N, 76.0842° E) Mysore district of Karnataka.	BDL
**KLS Tobacco 67**	Ramnathapura, (12.6166° N, 76.0842° E) Mysore district of Karnataka.	BDL
**KLS Tobacco 70**	Ramnathapura, (12.6166° N, 76.0842° E) Mysore district of Karnataka.	BDL
**KLS Tobacco 73**	Ramnathapura, (12.6166° N, 76.0842° E) Mysore district of Karnataka.	0.00389
**KLS Tobacco 80**	Ramnathapura, (12.6166° N, 76.0842° E) Mysore district of Karnataka.	0.00132
**KLS Tobacco 82**	Ramnathapura, (12.6166° N, 76.0842° E) Mysore district of Karnataka.	0.00897
**KLS Tobacco 84**	Ramnathapura, (12.6166° N, 76.0842° E) Mysore district of Karnataka.	0.00629
**KLS Tobacco 86**	Ramnathapura, (12.6166° N, 76.0842° E) Mysore district of Karnataka.	0.00498

*BDL=Below Detection Limit; NLS: Northern light soils; SLS; Southern Light Soils; KLS; Karnataka light soils.

Table 2. Pendimethalin residues present in soil samples analysed by GC-MS/SIM method.

**Sample Details**	**Location**	**Result**
BTJ S2	Podili, (15.6070° N, 79.6146° E) Prakasam district of Andhra Pradesh	BDL

BTJ S3	Kandukur, (15.2199° N, 79.9027° E) Prakasam district of Andhra Pradesh	BDL
BTJ S4	Jangareddygudem, (17.1223° N, 81.2923° E) West Godavari district of Andhra Pradesh	BDL
BTJ S7	Kaligiri, (14.8279° N, 79.6937° E) Prakasam district of Andhra Pradesh	BDL
BTJ S9	Koyyalagudem, (17.4521° N, 81.6528° E) West Godavari district of Andhra Pradesh	BDL
BTJ S10	Koyyalagudem, (17.4521° N, 81.6528° E) West Godavari district of Andhra Pradesh	BDL
BTJ S16	Koyyalagudem, (17.4521° N, 81.6528° E) West Godavari district of Andhra Pradesh	BDL
BTJ S21	Koyyalagudem, (17.4521° N, 81.6528° E) West Godavari district of Andhra Pradesh	BDL
BTJ S25	Kaligiri, (14.8279° N, 79.6937° E) Prakasam district of Andhra Pradesh	BDL
BTJ S27	Kaligiri, (14.8279° N, 79.6937° E) Prakasam district of Andhra Pradesh	BDL
BTJ S28	Kaligiri, (14.8279° N, 79.6937° E) Prakasam district of Andhra Pradesh	BDL
BTJ S40	Kaligiri, (14.8279° N, 79.6937° E) Prakasam district of Andhra Pradesh	BDL
BTJ S45	Kaligiri, (14.8279° N, 79.6937° E) Prakasam district of Andhra Pradesh	0.0002
BTJ S48	Kaligiri, (14.8279° N, 79.6937° E) Prakasam district of Andhra Pradesh	BDL
BTJ S50	Kaligiri, (14.8279° N, 79.6937° E) Prakasam district of Andhra Pradesh	0.0004
BTJ S29	Jangareddygudem, (17.1223° N, 81.2923° E) West Godavari district of Andhra Pradesh	0.0018
BTJ S30	Jangareddygudem, (17.1223° N, 81.2923° E) West Godavari district of Andhra Pradesh	0.00002
BTJ S49	Jangareddygudem, (17.1223° N, 81.2923° E) West Godavari district of Andhra Pradesh	BDL
BTJ S53	Jangareddygudem, (17.1223° N, 81.2923° E) West Godavari district of Andhra Pradesh	BDL
BTJ S54	Jangareddygudem, (17.1223° N, 81.2923° E) West Godavari district of Andhra Pradesh	BDL
BTJ S89	Podili, (15.6070° N, 79.6146° E) Prakasam district of Andhra Pradesh	0.0008
BTJ S90	Podili, (15.6070° N, 79.6146° E) Prakasam district of Andhra Pradesh	0.0001
BTJ S94	Podili, (15.6070° N, 79.6146° E) Prakasam district of Andhra Pradesh	BDL
BTJ S97	Podili, (15.6070° N, 79.6146° E) Prakasam district of Andhra Pradesh	BDL

*BDL=Below Detection Limit.

Table 3. Recovery percentage of pendimethalin in tobacco leaf and soil

Level of fortification (mg kg^{-1})	% Recovery	% RSD
	**Tobacco leaf**	
**0.01**	87.00	3.15
**0.02**	89.33	6.93
**0.05**	90.67	2.73
**0.1**	95.34	8.15
**0.5**	90.89	3.67
	**Soil**	

**0.01**	83.54	1.43
**0.02**	80.01	6.25
**0.05**	83.09	2.38
**0.1**	84.99	5.73
**0.5**	83.01	0.00

Figure Click here to access/download;Figure;Figures.docx

List of figures

Fig.1. Linearity calibration curve for solvent, tobacco leaf matrix matched and soil matrix matched standard.
Alt Text: Five concentration was taken to make the calibration curves.

Figure 2. Matrix effect during determination of pendimethalin, (a) pendimethalin response in solvent standard at 0.01 mg/kg (b) pendimethalin response in tobacco leaf matrix matched standard at 0.01 mg/kg (c) pendimethalin response in soil matrix matched standard at 0.01 mg/kg
Alt text: The analytical method developed for true detection of pendimethalin residue at very trace levels with acceptable recovery level and matrix effect

Figure 3. Different ion transition for quantification of pendimethalin in tobacco leaf matrix (a) 162, 191,208, 252& 281 ions (b) mass fragmentation pattern in Selection Ion Monitoring (SIM) mode
Alt text: GC-MS based selective ion monitoring (SIM) method has developed employing with different acquisition parameters such as confirmative identification based on the quantifier-qualifier ions (m/z) ratio

Figure 4. Different ion transition for quantification of pendimethalin in soil matrix
Alt text: Depending on the molecular fragmentation recorded in the mass detection system, four ions (*m/z*) were selected

Figure 5: Sample analysis for estimation of pendimethalin with the newly developed method, (a) sample with maximum pendimethalin residue present and (b) sample with BDL level of pendimethalin residue present
Alt text: The location specific variation of pendemethalin residue were analysed by the developed novel GC-MS method

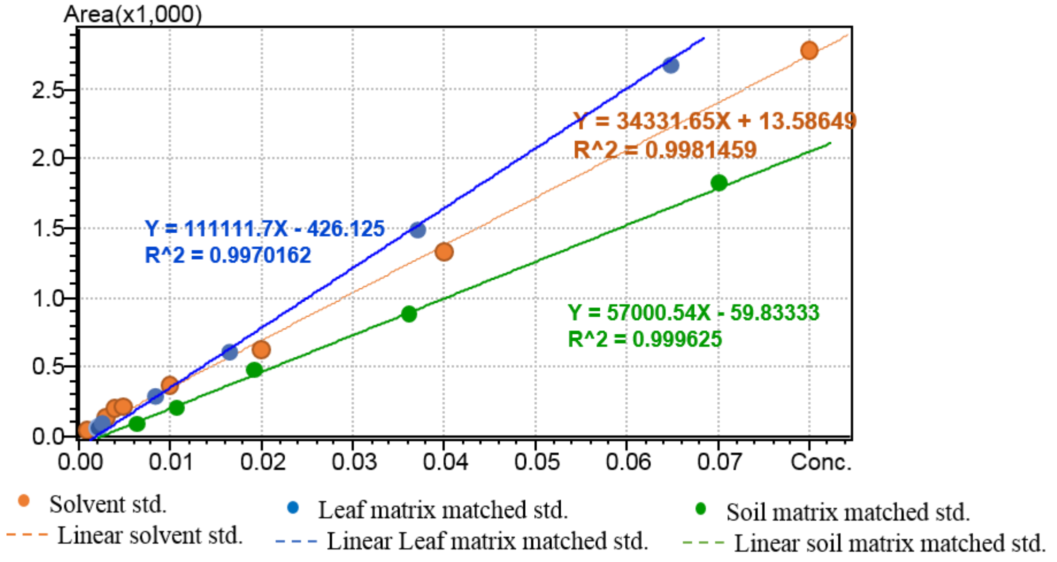

Fig.1. Linearity calibration curve for solvent, tobacco leaf matrix matched and soil matrix matched standard.

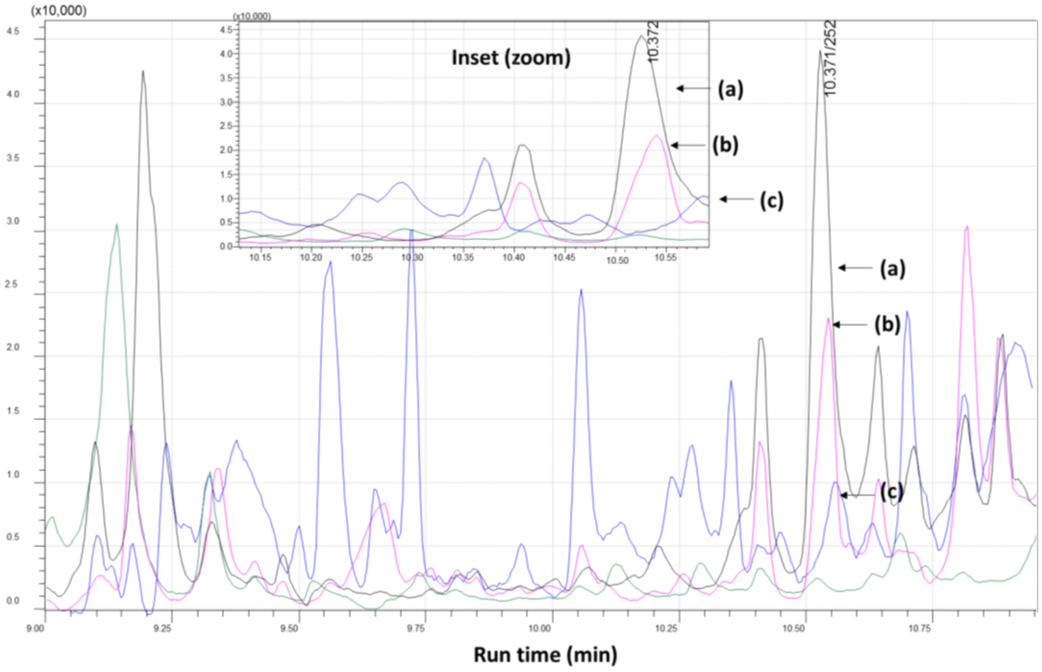

Figure 2. Matrix effect during determination of pendimethalin, (a) pendimethalin response in solvent standard at 0.01 mg/kg (b) pendimethalin response in tobacco leaf matrix matched standard at 0.01 mg/kg (c) pendimethalin response in soil matrix matched standard at 0.01 mg/kg

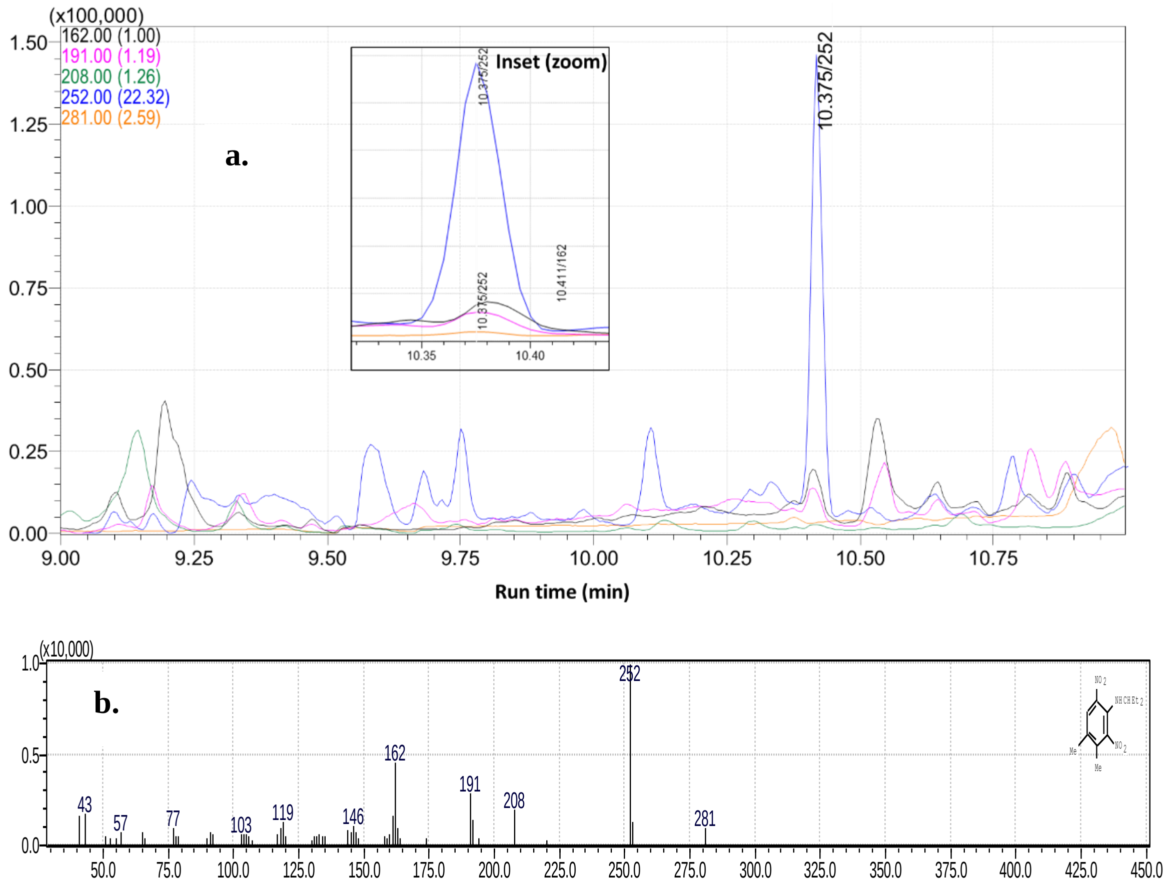

Figure 3. Different ion transition for quantification of pendimethalin in tobacco leaf matrix (a) 162, 191,208, 252& 281 ions (b) mass fragmentation pattern in Selection Ion Monitoring (SIM) mode

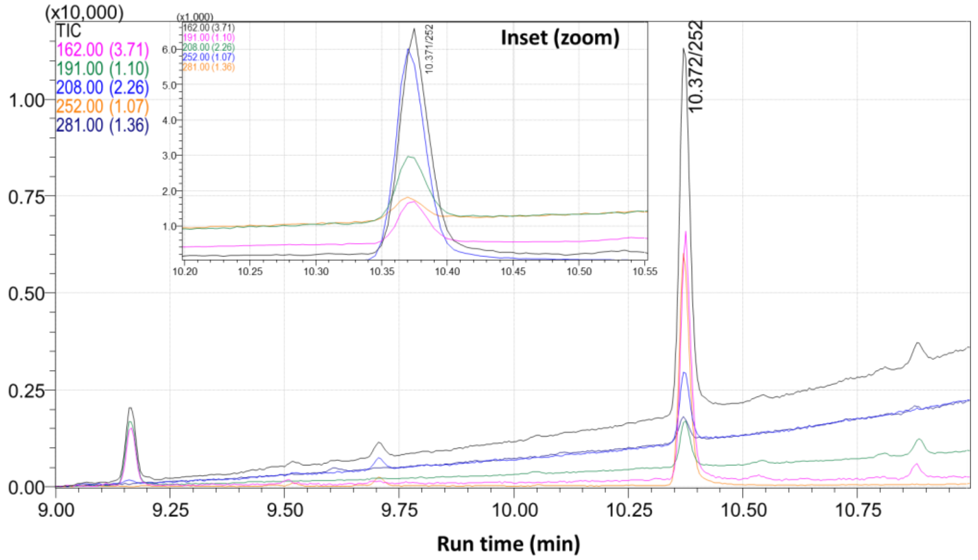

Figure 4. Different ion transition for quantification of pendimethalin in soil matrix

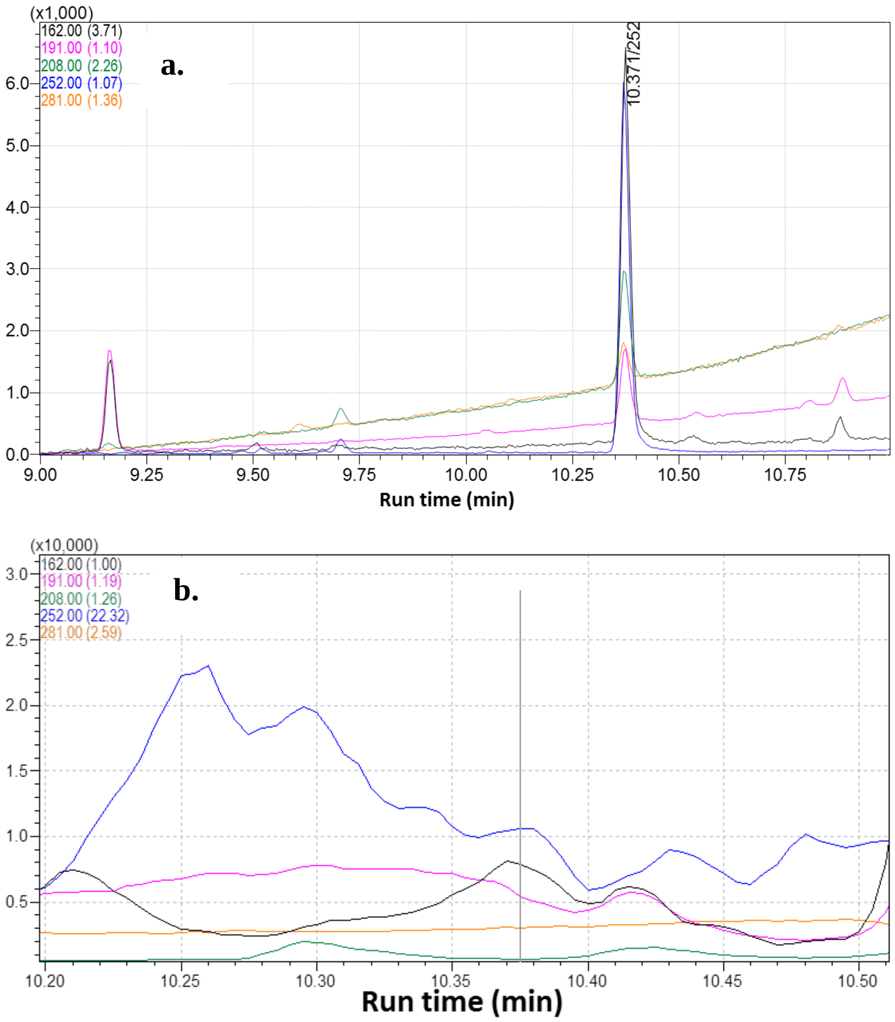

Figure 5: Sample analysis for estimation of pendimethalin with the newly developed method, (a) sample with maximum pendimethalin residue present and (b) sample with BDL level of pendimethalin residue present

Click here to access/download
Supporting Information
Supplimentar Figures.docx

Click here to access/download
Supporting Information - Compressed/ZIP File Archive
Supplementary Table.docx
