# Supplementary material for: An improved GC-MS-SIM analytical method for determination of pendimethalin residue in commercial crops (leaf and soils) and its validation
Source: PLoS One. 2025 Aug 28;20(8):e0328446. doi: 10.1371/journal.pone.0328446 (PMC12393745; doi:10.1371/journal.pone.0328446)
Supplement: Attachment — Submitted filename: Response to Reviewers.docx [file pone.0328446.s005.docx]

**Academic editor and Reviewers' comments**

PLOS One

| **Reviewers’ Comments** | **Authors’ Response** |
| --- | --- |
| **Reviewer:**   1. What kind of improvement of method has been done, mention in abstract. 2. L37-L39: Add values 3. L 53 Reference? 4. L106-L 107: Is it necessary? (After removing the midribs of the representative leaf samples, around 50 g of leaf lamina were oven dried at 60^o^ C for 2 h). 5. L 193: Add one or two lines why residue study is important in tobacco and soil. 6. L 206: Soil creates matrix effect? 7. L282: Reference 8. Table 2: Check the values; BTJS 45 & BTJS 90 | Thank you for your suggestion.  Added in abstract:  The method is improved in terms of sentivity and precision as per regulatory norms of exporting commercial crops like tobacco.  Added in line no. 38.  The method gave more than 80 % recovery (5 % RSD) showed compliance with international specification of DG-SANTE guidelines. In the majority of samples, pesticide residue levels were below the guidance residue levels (GRL; for pendimethalin GRL 5 ppm) value set by Center for Scientific Research Relative to Tobacco (CORESTA).  Added reference in Line no 56    We appreciate your comment  Yes, it is necessary to remove the midrib of tobacco leaf. As during manufacturing of FCV tobacco product to export only leaf lamina are used (by removing midrib), and thereby it is necessary to monitor pesticide residue only in leaf lamina. The leaf lamina to be dried to maintain the homogeneity of sample.  Added in Line no. 193:  Residue study in tobacco leaf and soil is highly essential for promoting residue management and ensure safe and rational use of pesticide in tobacco. It will also help for regulatory analysis of FCV tobacco which will promote the Indian tobacco exports in compliance with the acceptable levels of residues.  Thank you for the comment.  Yes. The soil also creates matrix effect. As The co-extractives present in soil matrix if co eluted in the final extract it may give the interference peak which affect the response of targeted pesticide, called as false negative.  Added the reference in Line no. 282  Thank you for your observation.  The values were rechecked and it is quantified at almost negligible values. |
